# The Theory-Practice Gap and the Impact of Reflection on Nursing Students’ Learning in the Practice Setting: A Scoping Review

**DOI:** 10.7759/cureus.117531

**Published:** 2026-10-06

**Authors:** Varvara Kakaidi, Konstantina Dimou, Maria Kitsiou, Agni Nakou, Maria Efstathiou, Elena Dragioti, Stefanos Mantzoukas

**Affiliations:** 1 Department of Nursing, School of Health Sciences, University of Ioannina, Ioannina, GRC; 2 Research Laboratory Psychology of Patients, Families, and Health Professionals, School of Health Sciences, University of Ioannina, Ioannina, GRC

**Keywords:** clinical practice, clinical supervision, nursing education, nursing students, reflective practice, simulation-based learning, theory–practice gap

## Abstract

The theory-practice gap remains a persistent challenge in nursing education, as nursing students often experience difficulties applying theoretical knowledge in real clinical settings. Reflective practice has been proposed as an educational strategy to facilitate the integration of theory and practice and enhance clinical learning. This review aimed to identify factors contributing to the theory-practice gap in nursing education and to examine the role of reflective practice in addressing this challenge. A systematic search was conducted in PubMed, Embase, and CINAHL (Cumulative Index to Nursing and Allied Health Literature) for studies published between 2013 and 2024. Quantitative, qualitative, and mixed-methods studies involving undergraduate nursing students during clinical placements were eligible for inclusion. A total of 38 studies met the inclusion criteria. The findings revealed a persistent discrepancy between theoretical instruction and clinical practice, with inadequate supervision, limited clinical support, insufficient preparation, and inconsistencies between educational and clinical environments identified as major contributing factors. Evidence derived predominantly from qualitative studies suggested that reflective practice, reflective journals, group discussions, mentorship, and structured clinical supervision may enhance critical thinking, self-confidence, professional identity formation, and the integration of theoretical knowledge into clinical practice, and contribute to narrowing the theory-practice gap and improving nursing students’ learning experiences. However, the implementation of reflective strategies remains inconsistent across nursing education programs. Further research is needed to evaluate the effectiveness of specific reflective interventions and educational models across different clinical settings.

## Introduction and background

Nurses working in hospital settings face complex challenges. Thus, the nursing profession demands a well-trained workforce that combines theoretical knowledge with practical skills. To deliver high-quality nursing care, competencies must be applied in accordance with evidence-based practice [1]. However, translating theory into practice remains challenging, especially for nursing students during clinical placements, in which many graduates experience difficulty applying their knowledge in complex situations. The theory-practice gap remains a persistent challenge in nursing education and highlights the need for educational strategies that support the effective integration of theoretical knowledge into clinical practice [2].

Theoretical frameworks are essential for guiding practice; however, they often fail to capture the complexity and dynamic nature of clinical environments. At the same time, clinical practice is influenced by practical constraints such as limited resources, time pressures, and the need for rapid decision-making [3]. These barriers restrict the effective application of theory in practice, highlighting the urgent need to address this disconnect in clinical education. A significant contributing factor is the predominant focus of nursing education on theoretical instruction at the expense of practical training. Consequently, many nursing students lack the competencies required to effectively apply theoretical knowledge in real clinical contexts. This issue is further exacerbated by professional nurses who, due to entrenched habits, limited confidence in contemporary methodologies, or insufficient training, may resist adopting innovative approaches [3]. This resistance perpetuates outdated practices and creates suboptimal role models for nursing students [4]. Furthermore, limited collaboration between academic institutions and clinical settings has resulted in theoretical approaches that are poorly aligned with clinical reality [2]. Leadership in nursing has often overlooked the need to bridge this gap, allowing the persistence of experience-based practices that lack strong scientific evidence [5].

Reflective practice is an educational approach that may contribute to addressing the theory-practice gap by promoting critical thinking, enhancing self-awareness, and supporting the integration of theoretical learning with clinical skills. It has gained recognition as a valuable strategy for bridging the gap between classroom learning and real-world clinical experiences. By encouraging nursing students to critically evaluate clinical situations, compare experiences with theoretical concepts, and identify areas for professional improvement, reflection facilitates active learning and the development of clinical reasoning skills [6,7]. Structured reflective activities also provide opportunities for feedback and self-assessment, which may enhance students’ confidence and ability to apply theoretical knowledge in complex clinical environments. In addition, other educational innovations, including simulation-based learning, the use of information and communication technologies (ICT), interactive e-learning, and interdisciplinary collaboration, have also been reported as useful approaches for preparing students for real-world nursing practice [8].

The theory-practice gap negatively affects nursing education, the professional development of graduates, and the quality of patient care. This phenomenon often reduces confidence among newly qualified nurses, contributes to frustration, and hinders professional development [2,9,10]. Thus, bridging the theory-practice gap is essential for developing competent professionals capable of delivering high-quality, patient-centered care. Reflective practice, combined with well-structured clinical placements, represents an important strategy for equipping nursing students with the skills required to manage the complexities of clinical environments [5].

This review aims to identify key challenges, effective strategies, and areas for future research, with the ultimate goal of advancing nursing education and practice. To achieve these objectives, the review was guided by the following research assumptions: a theory-practice gap exists in nursing education, and nursing students experience challenges in translating theoretical knowledge into clinical practice; appropriately structured clinical training may facilitate the integration of theory and practice by providing opportunities for experiential learning; and the incorporation of reflective practice into clinical education may support critical thinking, self-awareness, and confidence among nursing students.

## Review

Methods

Review Design

This was a scoping review, and the authors followed the Preferred Reporting Items for Systematic Reviews and Meta-Analyses Extension for Scoping Reviews (PRISMA-ScR) reporting guidelines to enhance methodological rigor and transparency; several methodological elements commonly used in systematic reviews, including duplicate screening procedures and methodological quality appraisal, were incorporated into the review process [11].

Population, Exposure, and Outcome

The study objectives were defined using the Population, Exposure, and Outcome (PEO) framework as follows: (i) Population: nursing students (undergraduate, pre-registered) enrolled in clinical placements, (ii) Exposure: participation in clinical placements, involvement in reflective practice, or studies exploring the theory-practice gap in nursing education, and (iii) Outcome: measures related to the application of theoretical knowledge in practice, the perceived or actual theory-practice gap, and the role of reflective practice in addressing this gap.

Search Strategy

To identify relevant studies, a comprehensive literature search was conducted in PubMed, Embase, and CINAHL (Cumulative Index to Nursing and Allied Health Literature). These databases were selected because they provide broad coverage of biomedical, nursing, and clinical education literature. The search strategy combined controlled vocabulary terms (e.g., MeSH (Medical Subject Headings) terms in PubMed) and free-text keywords related to nursing students, clinical placements, reflective practice, and the theory-practice gap, using the Boolean operators “AND” and “OR”. The final search was conducted in April 2025. The complete search strategy for all databases is provided in the Appendices. No additional manual or citation-tracking searches were systematically performed. These databases provide substantial coverage of the relevant literature, and additional databases such as Web of Science, Scopus, PsycINFO, and ERIC (Education Resources Information Center) were not searched; therefore, some potentially relevant studies may not have been identified.

Eligibility Criteria

Inclusion criteria: Primary research studies examining the theory-practice gap in nursing education, clinical placements, reflective practice, or educational interventions aimed at facilitating the integration of theoretical knowledge into clinical practice among nursing students were included. Quantitative (e.g., surveys, observational studies), qualitative (e.g., interviews, focus groups), and mixed-methods studies were considered. Only studies published in English between 2013 and 2024 were included. The starting year was selected to capture evidence reflecting contemporary nursing education models, clinical training environments, and professional competency frameworks relevant to current nursing practice. Although foundational literature on reflective practice and theory-practice integration predates 2013, the present review focused on evidence most applicable to current educational practices and clinical placement experiences.

Exclusion criteria: Articles were excluded if they focused on healthcare professionals other than nursing students or on students from other disciplines. Additionally, narrative reviews, literature reviews, and studies lacking a clear methodology were excluded. Editorials, opinion pieces, commentaries, and letters to the editor without original data were also excluded. No restrictions were applied regarding geographic location, clinical setting, or primary research methodology.

Study Selection

Two authors independently screened the titles and abstracts of all identified studies to assess relevance based on the inclusion and exclusion criteria. Full texts of potentially eligible studies were retrieved and reviewed independently by two authors to confirm eligibility. Studies that did not meet the eligibility criteria were excluded.

Quality Assessment

The authors used the Mixed Methods Appraisal Tool (MMAT), version 2018, to assess the methodological quality of the included studies [12]. The MMAT is a critical appraisal tool designed for the appraisal of qualitative, quantitative, and mixed-methods studies. The quality assessment was initially performed by one reviewer and subsequently verified by two additional members of the research team. Any discrepancies in scoring or interpretation were discussed and resolved through consensus. The MMAT checklist and criteria were used to evaluate study quality; however, no studies were excluded based solely on quality assessment results. Quality assessment was used to provide an overview of the methodological rigor of the included studies and to support interpretation of the findings.

Data Extraction

All included studies were imported into Zotero version 7 (Corporation for Digital Scholarship, Falls Church, Virginia, United States; https://www.zotero.org/blog/zotero-7/), and duplicates were removed. An Excel spreadsheet (Microsoft Corporation, Redmond, Washington, United States) was used to organize and screen the extracted data. The extracted variables included the PubMed identifier (PMID) and/or digital object identifier (DOI), journal name, article title, first author’s name, year and country of publication, study design, sample characteristics (including mean age and gender), and key findings. Data extraction was conducted by one author and verified by two others to ensure accuracy and consistency.

Data Synthesis

Due to the heterogeneity of study designs, outcome measures, and reported findings, a quantitative synthesis or meta-analysis was not considered appropriate. The findings of the included studies were synthesized narratively using a thematic approach. Studies were reviewed and grouped according to recurring themes related to the theory-practice gap, clinical learning environments, mentorship and support, reflective practice, simulation-based learning, and strategies for improving clinical education.

Results

Search Results

The authors identified 1,370 records through database searching (PubMed, CINAHL, and Embase) and an additional 44 records through manual searching, resulting in a total of 1414 records. After removing duplicates (n = 210), 1,204 records remained for title and abstract screening. Following screening, 1,108 records were excluded. Full-text articles were sought for retrieval (n = 96), of which nine could not be retrieved. A total of 87 full-text articles were assessed for eligibility, and 49 reports were excluded for predefined reasons. Ultimately, 38 studies met the inclusion criteria and were included in the final review (Figure 1) [2-4,6,9,10,13-44].

**Figure 1:**
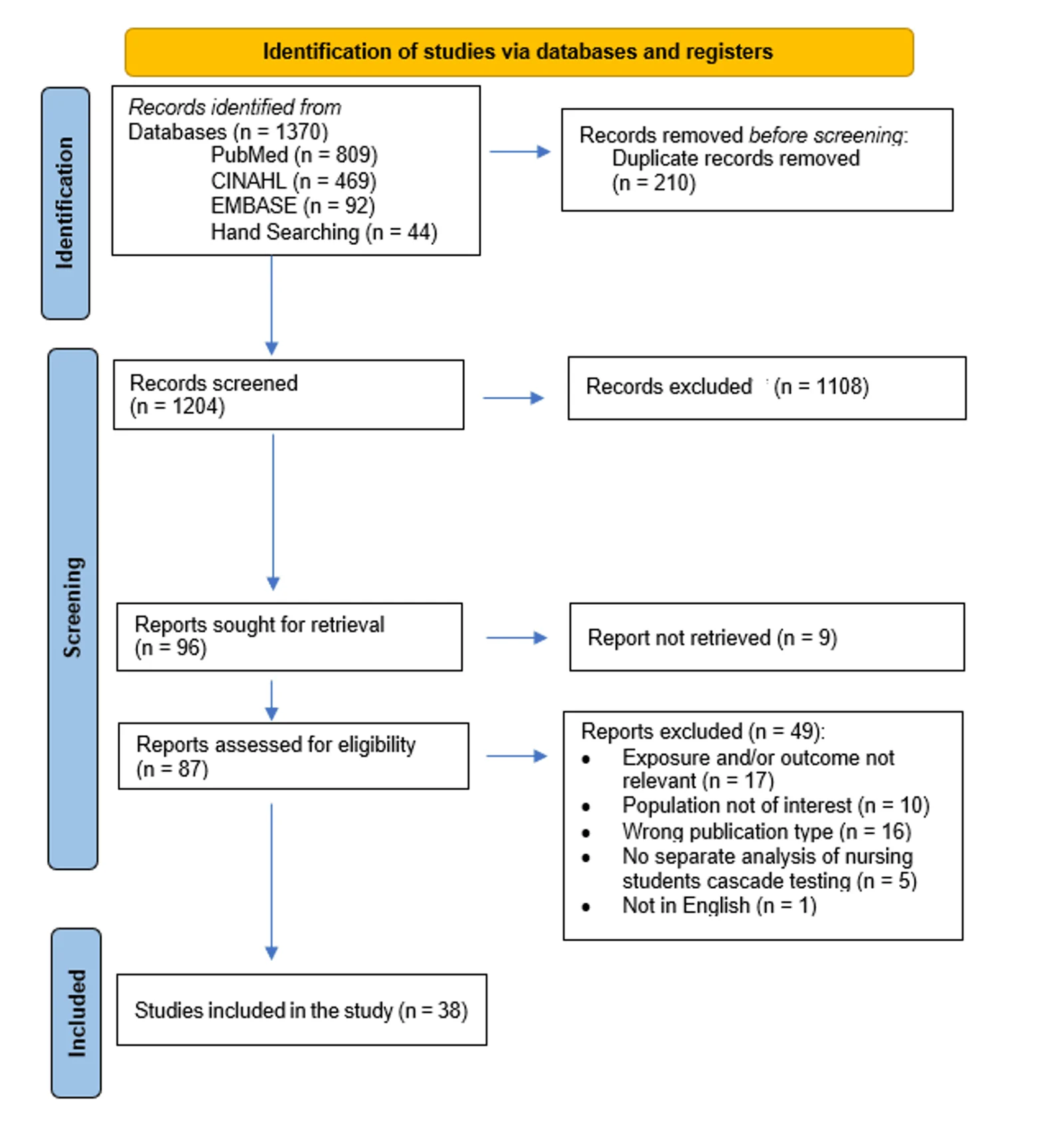
PRISMA-ScR flow diagram of the study selection process PRISMA-ScR: Preferred Reporting Items for Systematic reviews and Meta-Analyses-Scoping Review

Characteristics of the Included Studies

Table 1 summarizes the characteristics of the included studies. A total of 38 studies met the inclusion criteria and were published between 2013 and 2024. Seven studies were conducted in European countries [19,25,26,28,31,36,41], while the remaining studies were conducted across diverse international educational and healthcare settings. A total of 32 studies (84.2%) were qualitative [3,4,6,9,10,12,15-39,43], four (10.5%) were quantitative [2,13,40,41], and two (5.3%) used mixed methods [14,42]. The predominance of qualitative research suggests that the current evidence base is largely derived from nursing students' perceptions and experiences of clinical education and the theory-practice gap. The total number of participants across the included studies was 2,091, including 469 (22.5%) male and 1,622 (77.5%) female participants, with a mean age of 26.8 years.

**Table 1:** Characteristics of the included studies TPSN: teacher, patient, student, nurse; NR: not reported

Author (Year)	Country	Study Design	Mean Age (years)	Key Findings
Ramsbotham et al. (2019) [2]	Vietnam	Quantitative	20.9	Longer clinical placements were associated with higher-quality clinical learning experiences
Vosoughi et al. (2022) [3]	Iran	Qualitative	NR	The TPSN collaborative learning model improved participation, communication, and self-confidence in clinical practice
Saifan et al. (2021) [4]	United Arab Emirates	Qualitative	22	Students emphasized curriculum reform, reflective learning, and stronger integration of theory and practice
Saifan et al. (2021b) [6]	United Arab Emirates	Qualitative	21.5	Students perceived a persistent gap between theoretical instruction and clinical practice
Jasemi et al. (2018) [9]	Iran/UK	Qualitative	NR	Ineffective teaching and poor interactions hindered clinical learning
Wallace (2016) [10]	United States	Qualitative	NR	Consistent supervision and structured clinical placements enhanced students’ clinical learning experiences
Ahmadi et al. (2020) [13]	Iran	Qualitative	22.5	Barriers included lack of support, poor infrastructure, and unfair evaluation
Alrazeeni et al. (2021) [14]	Saudi Arabia/Canada/Pakistan	Quantitative	22	Clinical skills improved significantly during internship compared to practicum
Anderson et al. (2015) [15]	Australia	Mixed methods	32	Evidence-based practice viewed as essential to professional development
Asadizaker et al. (2015) [16]	Iran	Qualitative	NR	Effective clinical education requires planning, support, and collaboration
Ashipala et al. (2023) [17]	Namibia	Qualitative	30	Clinical exposure enhances learning, but limited resources hinder training
Baraz et al. (2014) [18]	Iran	Qualitative	21	Learning occurs through observation, engagement, and communication
Barisone et al. (2019) [19]	Italy/Ireland	Qualitative	23.6	Multimedia learning improves clinical skills and communication
Bawadi et al. (2019) [20]	Jordan	Qualitative	33	Inequality, limited resources, and theory–practice mismatch affect learning
Bazrafkan and Kalyani (2018) [21]	Iran	Qualitative	NR	Lack of instructor support reduces motivation and learning
Botma (2014) [22]	South Africa	Qualitative	NR	Simulation enhances confidence, teamwork, and theory–practice integration
Calleja et al. (2016) [23]	Australia	Qualitative	NR	Positive relationships and preparation improve clinical learning
Factor et al. (2017) [24]	Philippines	Qualitative	19.5	Lack of resources and poor role models hinder clinical learning
Falk et al. (2016) [25]	Sweden	Qualitative	26	Supervisor interaction improves integration of theory and practice
Günay & Kılınç (2018) [26]	Turkey	Qualitative	20.8	Limited practice, poor support, and communication issues reduce confidence
Günaydin & Çoban (2021) [27]	Turkey	Qualitative	NR	Anxiety and lack of preparation hinder clinical learning
Houghton et al. (2013) [28]	Ireland	Qualitative	NR	Simulation preferred, but real practice remains challenging
Jahanpour et al. (2016) [29]	Iran	Qualitative	22.5	Positive mentorship improves confidence and learning
Kalogirou et al. (2021) [30]	Canada	Qualitative	>18	Curriculum redesign and stronger academic–practice collaboration were considered essential to reduce the theory–practice gap
Marañón & Pera (2015) [31]	Spain	Qualitative	NR	Clinical placements and reflective learning supported professional identity development and integration of theory and practice
Muthathi et al. (2017) [32]	South Africa	Qualitative	NR	Small-group demonstrations and consistent clinical facilitation enhanced skills learning and confidence
Ngozika et al. (2023) [33]	Nigeria	Qualitative	27	Negative staff attitudes and poor clinical learning environments contributed to the theory–practice gap
Rahmati Sharghi et al. (2015) [34]	Iran	Qualitative	28.2	Limited practical exposure, inadequate infrastructure, and insufficient clinical preparation hindered competency development
Salifu et al. (2019) [35]	Ghana	Qualitative	25.5	Poor program design, limited supervision, and inadequate resources contributed to the theory–practice gap
Stoffels et al. (2022) [36]	Netherlands	Qualitative	22	Differences between educational and clinical settings reduced confidence and challenged theory–practice integration
Waller & Nestel (2019) [37]	Australia	Qualitative	27	Interprofessional simulation and reflective practice enhanced collaboration and clinical learning
Willemse (2015) [38]	South Africa	Qualitative	NR	WhatsApp-supported peer interaction improved communication, support, and learning experiences
Yousefy et al. (2015) [39]	Iran	Qualitative	23.1	Role ambiguity, routine-based care, and inadequate clinical support contributed to the theory–practice gap
Zamanzadeh et al. (2024) [40]	Iran	Qualitative	23	Lack of support, inadequate infrastructure, and unclear professional roles hindered clinical education experiences
Koldestam et al. (2024) [41]	Sweden	Quantitative	26.3	Reflective learning and holistic supervision were considered essential for meaningful clinical learning
Şimşek et al. (2023) [42]	Turkey	Quantitative	21.16	Many students reported uncertainty in clinical practice due to the theory–practice gap
Woo & Newman (2020) [43]	Singapore	Mixed methods	27.5	Transition to professional practice was perceived as stress
Safazadeh et al. (2018) [44]	Iran	Qualitative	NR	Lack of professional awareness, limited critical thinking, and inadequate practical preparation contributed to the theory–practice gap

Study Quality

Table 2 presents the methodological quality assessment of the included studies using the 2018 version of the MMAT. Overall, the methodological quality of the included evidence was generally high. Thirty-two qualitative studies [3,4,6,9,10,12,15-39,43] and four quantitative studies [2,13,40,41] were rated as high quality, meeting the majority of the MMAT criteria. The two mixed-methods studies were rated as moderate quality due to unclear reporting regarding the integration of qualitative and quantitative findings, particularly in relation to addressing divergences and inconsistencies between study components. The predominance of high-quality studies increases confidence in the main themes identified across the review. However, findings derived from studies with methodological limitations were interpreted cautiously. 

**Table 2:** Methodological quality assessment of included studies using the Mixed Methods Appraisal Tool (MMAT) 2018

Study (Author, Year)	Study Design	MMAT Score	Overall Quality
Ahmadi et al. (2020) [13]	Qualitative	5/5	High
Asadizaker et al. (2015) [16]	Qualitative	5/5	High
Ashipala et al. (2023) [17]	Qualitative	5/5	High
Baraz et al. (2014) [18]	Qualitative	5/5	High
Barisone et al. (2019) [19]	Qualitative	5/5	High
Bawadi et al. (2019) [20]	Qualitative	5/5	High
Bazrafkan & Kalyani (2018) [21]	Qualitative	5/5	High
Botma (2014) [22]	Qualitative	5/5	High
Calleja et al. (2016) [23]	Qualitative	5/5	High
Factor et al. (2017) [24]	Qualitative	5/5	High
Falk et al. (2016) [25]	Qualitative	5/5	High
Günay & Kılınç (2018) [26]	Qualitative	5/5	High
Günaydin & Çoban (2021) [27]	Qualitative	5/5	High
Houghton et al. (2013) [28]	Qualitative	5/5	High
Jahanpour et al. (2016) [29]	Qualitative	5/5	High
Jasemi et al. (2018) [9]	Qualitative	5/5	High
Kalogirou et al. (2021) [30]	Qualitative	5/5	High
Marañón & Pera (2015) [31]	Qualitative	5/5	High
Muthathi et al. (2017) [32]	Qualitative	5/5	High
Ngozika et al. (2023) [33]	Qualitative	5/5	High
Rahmati Sharghi et al. (2015) [34]	Qualitative	5/5	High
Safazadeh et al. (2018) [44]	Qualitative	5/5	High
Saifan et al. (2021a) [6]	Qualitative	5/5	High
Saifan et al. (2021b) [4]	Qualitative	5/5	High
Salifu et al. (2019) [35]	Qualitative	5/5	High
Stoffels et al. (2022) [36]	Qualitative	5/5	High
Vosoughi et al. (2022) [3]	Qualitative	5/5	High
Wallace (2016) [10]	Qualitative	5/5	High
Waller and Nestel (2019) [37]	Qualitative	5/5	High
Willemse (2015) [38]	Qualitative	5/5	High
Yousefy et al. (2015) [39]	Qualitative	5/5	High
Zamanzadeh et al. (2024) [40]	Qualitative	5/5	High
Alrazeeni et al. (2021) [14]	Quantitative	5/5	High
Koldestam et al. (2024) [41]	Quantitative	5/5	High
Ramsbotham et al. (2019) [2]	Quantitative	5/5	High
Şimşek et al. (2023) [42]	Quantitative	4/5	High
Anderson et al. (2015) [15]	Mixed methods	4/5	Moderate
Woo & Newman (2020) [43]	Mixed methods	4/5	Moderate

The Gap Between Nursing Education and Clinical Practice

Relationship between theory and practice: The included studies generally suggested that gender was not a major determinant of students' clinical placement experiences. In contrast, the year of study and duration of clinical placement appeared to influence learning outcomes, with students reporting more positive learning experiences when clinical placements extended beyond four weeks [2]. Across studies, a recurring theme was the difficulty of translating theoretical knowledge into clinical practice, particularly in unfamiliar or highly specialized settings [4,27]. Feelings of anxiety, uncertainty, and inadequate preparation were commonly reported, especially in psychiatric placements and situations where educational objectives were perceived as unclear [2,27]. Several studies also highlighted perceived inequalities in learning opportunities, with some nursing students reporting that medical students were prioritized within clinical environments, limiting opportunities for practical skill development and contributing to feelings of neglect [20]. 

Clinical education in nursing: The active participation of both instructors and students plays a crucial role in ensuring effective clinical education. Instructors must collaborate closely with clinical settings to enhance the overall learning experience and improve the outcomes of clinical practice [3]. However, a significant challenge observed among students is low self-confidence, which often makes them feel inadequate in performing clinical skills in real-life scenarios. This lack of confidence acts as a barrier to patient interaction, as students fear making mistakes and providing incorrect nursing care [26]. Additionally, many students struggle to transfer theoretical knowledge into clinical practice. This difficulty is largely attributed to the increased workload during clinical training, which overwhelms students and limits their ability to fully integrate theoretical concepts into real-world applications [27].

Interaction with clinical staff: Several included studies reported variability in students' experiences with clinical staff during placements. While some students described supportive and respectful interactions, others reported dismissive or unsupportive behaviors, which were perceived as negatively affecting communication, confidence, and learning experiences within the clinical environment. Many students emphasized that positive relationships with clinical staff greatly enhanced their learning, making them feel valued and more engaged in their training. Conversely, some nurses treated students as insignificant, which led to feelings of discouragement, reduced motivation for practical training, and even doubts about their career choice in nursing [13,26]. Additionally, students often felt overwhelmed by the excessive demands placed on them by nursing staff. This intensified their fear of making mistakes and made them hesitant to ask questions or express concerns, further hindering their ability to learn effectively [4].

Lack of support in clinical training: Several students claimed that they felt inadequately prepared for clinical practice due to the training they had received up to that point [35,40]. Some expressed that they were overwhelmed by feelings of anxiety and confusion, especially when they had to deal with difficult situations in the clinical setting, such as conflicts or complaints. This sense of inadequate preparation gave them the feeling that they can easily be led astray. At the same time, they felt uncertain about safely handling difficult situations in the clinical setting [13]. Additionally, a significant number of students felt a sense of exclusion in the clinical environment. The lack of designated break areas, inadequate storage for personal belongings, and the absence of appropriate changing facilities were daily difficulties that hindered their adaptation and the educational process. Additionally, they also reported difficulties accessing the designated training space because the administration did not provide them with the appropriate access cards. Thus, students wait for a considerable amount of time outside the clinic, directly resulting in lost training hours [26]. While public hospitals provide greater independence in the education of students, there is concern about the reduced supervision, which creates educational uncertainty regarding decision-making and the application of skills [20].

Correct Organization of Clinical Exercise and the Positive Effect on Bridging the Gap

Factors positively influencing clinical practice: Students often experience anxiety when about to start clinical practice because they feel they lack the necessary knowledge and experience to provide care to patients. Additionally, they experience anxiety about how to manage the various cultural backgrounds in the hospital setting as well as the potential complex medical situations that may arise. Adequate preparation was identified as an important prerequisite for the successful commencement of clinical training [40]. Despite any concerns, clinical practice in real-world settings contributes to the connection between theory and practice and the substantial learning of practical skills. Clinical practice in hospital settings allows students to acquire new knowledge and better understand the clinics through information seeking [31]. A structured educational program with appropriate educational content maximizes the benefits of clinical education. In addition, specialized human resources and supportive interactions between nursing staff and students may contribute positively to the clinical learning experience. Effective patient assessment may be facilitated when communication between students and clinical staff is strong, leading to greater student satisfaction [16]. The creation of smaller clinical groups allows students to observe skills more attentively, thus gaining more opportunities for practical training. Many argue that laboratory exercises need to be directly aligned with clinical skills because they would contribute to the reinforcement and retention of knowledge, build confidence, and facilitate the transition to real practice [15,32]. Learning skills in a simulated laboratory environment does not suit all students, so some prefer to learn skills directly in the clinical setting [28]. Finally, students also recognize that evidence-based nursing is a critical element in the learning process, as it enhances professional identity and clinical competence [15,32].

Learning and communication strategies: The included studies suggest that when students observe clinical skills performed by experienced nurses. Thus, their confidence in clinical practice is strengthened, and they establish correct nursing techniques. Additionally, good communication with peers and discussions with fellow students are factors that contribute to substantial learning and consequently to the enhancement of self-confidence. The anxiety of clinical practice decreases, and overall performance improves when practical training has been conducted before clinical practice [32]. An important factor in enhancing the clinical experience was the strong level of collaboration between supervisors and nursing staff. This collaborative environment provided better guidance and support, leading to a more effective learning process [33].

Structured clinical education and training enhancement: Many nursing students struggle to apply theoretical knowledge when transitioning from laboratory training to real patient care. The lower the year of study, the more pronounced this challenge becomes, as younger students often have limited clinical experience and underdeveloped critical thinking skills. Students expressed differing views and remain divided on whether they would perform better in clinical settings if their knowledge had been reinforced through laboratory simulations or real-world practice [4]. A structured and holistic approach to clinical education appears to help bridge the gap between theory and practice. The TPSN (teacher, patient, student, nurse) model, for example, focuses on the healthcare recipient and integrates guidance, prior experience, and comprehensive clinical education, making it an effective framework for improving practical training [3].

Incorporating Reflective Practice into Clinical Practice

Reflection in clinical learning: Across the included studies, reflective practice emerged as an important strategy for supporting the integration of theoretical knowledge into clinical learning [30,44]. Reflection was reported to facilitate critical thinking, self-awareness, and the analysis of clinical experiences, enabling students to connect classroom learning with real-world practice [26,31,45]. Reflective activities included guided discussions, feedback from clinical supervisors, and structured consideration of previous clinical experiences [4,31,45]. Several studies suggested that reflective practice may contribute to increased self-confidence, professional identity formation, and clinical decision-making skills [26,31]. Students frequently reported that opportunities to reflect on clinical experiences helped them identify learning needs, recognize strengths and weaknesses, and better understand the application of theoretical concepts within clinical settings [26,31,45]. Clinical mentors and supervisors were described as important facilitators of reflective learning through guidance, feedback, and supportive discussion [4,31].

Other Factors that Contribute to Bridging the Gap Between Theory and Practice

Role of the clinical consultant and mentorship: Several studies suggested that clinical supervisors should be experienced nurses who also take on the role of a clinical consultant. The contribution of the clinical advisor extends beyond the teaching of clinical skills; their most important contribution focuses on shaping the professional identity of the students and imparting the fundamental values of nursing. Students claimed that the active presence of the mentor in the learning process is a significant element of the learning process [31].

Conversely, when the clinical advisor exhibits negative behavior towards the students or the students are afraid to express questions that arise in the clinical setting, the learning process does not progress [33]. An organized and supportive educational framework, supported by clinical mentors who provide guidance and constructive feedback, may help bridge the gap between theory and practice [26]. Guidance and feedback were also associated with increased self-confidence and improved communication between students, mentors, and the clinical environment [27].

Clinical mentors with strong theoretical knowledge and extensive clinical experience were frequently described as important role models for students [29]. In addition, reducing mentors’ workload has been proposed as a strategy to facilitate the provision of effective guidance and feedback [39]. Overall, effective and high-quality supervision may contribute to a more coherent and enriched educational experience [25].

Role of simulation in education: Simulation-based learning was frequently reported as a valuable educational strategy for supporting the transition from theoretical learning to clinical practice [22,37]. Students highlighted the opportunity for active participation and skills development within a safe learning environment, which may enhance confidence and preparedness for real-world clinical situations [22]. Interprofessional simulation and patient-centered learning models were also described as approaches that may support collaboration, engagement, and clinical learning experiences [37].

Opinions on evaluation: Students reported that both academic staff and hospital-based supervisors were involved in the evaluation process. However, concerns regarding the fairness and objectivity of assessment were frequently expressed. Some students believed that supervisors did not have a complete understanding of their clinical performance because of limited direct observation during placements. In contrast, students felt that ward nurses who worked more closely with them were often better positioned to evaluate their progress and development [13].

The methods used for assessment were also questioned. Some students expressed reservations about the use of written assignments as evaluation tools and suggested that direct observation of clinical skills provided a more accurate reflection of competence and learning outcomes. In addition, a number of students perceived that certain clinical nurses contributed little to the educational process and, in some cases, negatively influenced evaluation outcomes [26,40].

Discussion

In this scoping review, several important factors were identified that may contribute to effective clinical education and help bridge the gap between theory and practice. 

Integration of Theoretical Knowledge into Clinical Practice

The integration of theoretical knowledge into clinical practice remains one of the most persistent challenges in nursing education. The findings of this review suggest that the theory-practice gap is influenced by multiple educational, organizational, and personal factors rather than a single cause. Students frequently reported difficulties in translating classroom knowledge into real-world clinical practice, particularly when learning objectives were unclear or when clinical environments did not adequately support learning opportunities [27].

A recurring theme across the included studies was the mismatch between theoretical preparation and the realities of clinical practice. This discrepancy may contribute to uncertainty, reduced confidence, and reliance on rote memorization rather than meaningful learning [46]. Similar observations have been reported in previous nursing education literature, where inadequate integration between academic and clinical learning environments has been identified as a barrier to professional development and clinical competence.

The findings also highlight the importance of educational design in supporting the transition from theory to practice. Structured clinical programs with clearly defined learning objectives and opportunities for supervised practice may facilitate knowledge integration and improve communication between students and clinical staff [3,16]. Furthermore, laboratory-based clinical exercises and skills training were frequently perceived by students as useful preparatory experiences that may enhance confidence and readiness for clinical placements [15,32].

Taken together, these findings suggest that reducing the theory-practice gap requires not only curricular improvements but also stronger alignment between academic instruction and clinical learning environments. Creating educational experiences that promote active learning, structured supervision, and progressive skill development may support nursing students in translating theoretical knowledge into safe and effective clinical practice. 

The Importance of the Role of Clinical Staff and Support Systems

The findings of this review highlight the central role of clinical staff, mentors, and supervisors in shaping nursing students' learning experiences. Supportive and respectful interactions with clinical staff were consistently associated with greater confidence, engagement, and perceived learning opportunities, whereas dismissive or unsupportive behaviors were reported as barriers to communication, participation, and professional development [13,26].

These findings reinforce previous evidence emphasizing the importance of mentorship and supportive supervision within clinical education [40]. Clinical mentors not only facilitate the acquisition of practical skills but also help students navigate unfamiliar clinical environments, develop professional identity, and build confidence in their clinical decision-making abilities. Constructive feedback and opportunities for guided learning appear particularly important in supporting the transition from theoretical learning to clinical practice.

The findings further suggest that the quality of supervision may be influenced by organizational factors, including staff workload and the availability of clinical educators. Although the included studies did not directly evaluate optimal supervision models, students frequently emphasized the value of accessible mentors and individualized support. Therefore, educational strategies that promote effective mentorship, constructive feedback, and supportive learning environments may contribute to more positive clinical learning experiences.

The Importance of the Clinical Environment and the Duration of Clinical Practice

The findings of this review suggest that both the clinical environment and the duration of clinical placements may influence students' learning experiences and perceived preparedness for professional practice. Some studies reported that longer clinical placements were associated with greater familiarity with clinical routines, increased confidence, and more opportunities to apply theoretical knowledge in practice [2]. However, the available evidence remains limited, and the optimal duration of clinical placements warrants further investigation.

The review also highlighted the importance of contextual factors within clinical settings. Students undertaking placements in specialized environments, such as psychiatric units, frequently reported higher levels of stress and uncertainty, often attributing these experiences to limited preparation for specialized clinical contexts [27]. These findings suggest that targeted preparatory activities and orientation programs may help students adapt more effectively to unfamiliar clinical environments.

In addition, some students perceived inequalities in access to learning opportunities, particularly when nursing students felt less prioritized than medical students within clinical settings [20]. Such experiences may reduce engagement and limit opportunities for skills development. Promoting equitable access to clinical experiences and learning opportunities may therefore contribute to a more supportive and inclusive educational environment.

The Importance of Reflective Practice and Simulation

Reflective practice emerged as one of the most frequently discussed educational approaches identified in this review. The included studies suggest that reflection may facilitate the integration of theoretical knowledge into clinical practice by encouraging students to critically evaluate their experiences, identify learning needs, and connect classroom learning with real clinical situations [3,31,45]. In addition to supporting critical thinking, reflective practice was associated with greater self-awareness, confidence, and professional development. Guided reflective approaches, including structured reflection models such as TPSN, may be particularly valuable because they provide students with opportunities to receive feedback and systematically analyze clinical experiences [3].

The findings also suggest that reflective practice may contribute to bridging the theory-practice gap by promoting active learning and encouraging students to engage more deeply with clinical experiences. However, the implementation of reflective practice varied considerably across studies, and limited information was available regarding the frequency, duration, and specific formats of reflective activities. Further research is needed to determine which reflective approaches are most effective in supporting learning outcomes across different educational contexts.

Simulation-based education was also frequently described as a useful educational strategy. Students reported that simulation may provide a safe and supportive learning environment in which theoretical concepts can be applied and clinical skills practiced before exposure to real clinical situations [22,37]. Patient-centered simulation models, such as Simulated Client Interprofessional Education (SCIPE), were reported to support engagement and practical skill development [37]. Nevertheless, the available evidence remains largely qualitative, and further comparative studies are needed to evaluate the impact of simulation-based interventions on long-term educational outcomes.

Suggestions for Improving Clinical Education

The findings of this review suggest several potential strategies that may help reduce the theory-practice gap in nursing education. First, several studies suggested that longer clinical placements may provide students with greater opportunities to develop confidence, apply theoretical knowledge, and become familiar with clinical routines [2]. However, further research is needed to determine the optimal duration of clinical placements across different educational and clinical contexts.

The review also highlights the potential value of preparatory programs and simulation-based learning activities. These approaches may support students' readiness for clinical practice by providing opportunities to develop clinical skills and apply theoretical concepts within structured learning environments [22,37].

In addition, fostering supportive and collaborative learning environments may contribute to more positive educational experiences. Positive interactions between students, clinical staff, and supervisors were frequently associated with increased engagement, confidence, and perceived learning opportunities [13,26]. Reflective practice also emerged as a promising educational approach, as it may support critical thinking, self-awareness, and the integration of theoretical knowledge into clinical practice [3,31,45].

Finally, the findings suggest that clear learning objectives, effective supervision, and supportive clinical environments may facilitate students' transition from theory to practice. Nevertheless, the effectiveness of these strategies should be evaluated further through robust educational research conducted across diverse clinical and educational settings.

## Conclusions

The integration of theoretical knowledge into clinical practice remains a persistent challenge in nursing education. The findings of this scoping review suggest that nursing students frequently encounter difficulties in applying theoretical concepts within clinical settings, particularly when learning objectives are unclear, supervision is inadequate, or learning opportunities are unevenly distributed. Factors such as workload, clinical learning environments, and the quality of educational support may influence students' learning experiences and their ability to translate knowledge into practice. The available evidence also suggests that supportive relationships with clinical staff, mentors, and academic supervisors are important for fostering confidence, professional identity development, and engagement in learning.

Reflective practice emerged as a promising educational approach that may support critical thinking, self-awareness, and the integration of theoretical knowledge into clinical experiences. Similarly, simulation-based learning was frequently described as a useful strategy for preparing students for clinical practice within a safe learning environment. Overall, the findings suggest that well-structured clinical education programs, supportive learning environments, reflective learning opportunities, and effective collaboration between academic and clinical educators may help reduce the theory-practice gap. However, the evidence base remains heterogeneous and is predominantly qualitative. Further research is needed to evaluate the effectiveness of specific educational interventions, including reflective and simulation-based approaches, across diverse educational and clinical contexts.

## Appendices

Database search strategy 

**Table 3:** Full search strategy for databases CINAHL: Cumulative Index to Nursing and Allied Health Literature

Database	Search Strategy
PubMed	("nursing students" OR "student nurses") AND ("theory-practice gap" OR "theory practice gap") AND ("clinical practice" OR "clinical placement") AND (reflection OR reflective practice)
CINAHL	("nursing students") AND ("clinical education") AND ("theory-practice gap") AND ("reflective learning")
EMBASE	('nursing student'/exp OR 'student nurse') AND ('clinical practice'/exp OR 'clinical placement') AND ('theory-practice gap') AND ('reflection'/exp OR 'reflective practice')
